# TOMOBFLOW: feature-preserving noise filtering for electron tomography

**DOI:** 10.1186/1471-2105-10-178

**Published:** 2009-06-12

**Authors:** Jose-Jesus Fernandez

**Affiliations:** 1 Dept. Computer Architecture and Electronics, University of Almería, 04120 Almería, Spain; 2 Centro Nacional de Biotecnologia – CSIC, Campus de Cantoblanco, 28049 Madrid, Spain

## Abstract

**Background:**

Noise filtering techniques are needed in electron tomography to allow proper interpretation of datasets. The standard linear filtering techniques are characterized by a tradeoff between the amount of reduced noise and the blurring of the features of interest. On the other hand, sophisticated anisotropic nonlinear filtering techniques allow noise reduction with good preservation of structures. However, these techniques are computationally intensive and are difficult to be tuned to the problem at hand.

**Results:**

TOMOBFLOW is a program for noise filtering with capabilities of preservation of biologically relevant information. It is an efficient implementation of the Beltrami flow, a nonlinear filtering method that locally tunes the strength of the smoothing according to an edge indicator based on geometry properties. The fact that this method does not have free parameters hard to be tuned makes TOMOBFLOW a user-friendly filtering program equipped with the power of diffusion-based filtering methods. Furthermore, TOMOBFLOW is provided with abilities to deal with different types and formats of images in order to make it useful for electron tomography in particular and bioimaging in general.

**Conclusion:**

TOMOBFLOW allows efficient noise filtering of bioimaging datasets with preservation of the features of interest, thereby yielding data better suited for post-processing, visualization and interpretation. It is available at the web site .

## Background

The advent of bioimaging technology has made it possible to observe the molecular and cellular architecture and interactions that underlie essential functions within cells and tissues. The availability of bioimaging techniques (e.g. light, confocal, X-ray, electron microscopies) in laboratories is growing rapidly. So is the need for advanced image processing methods that facilitate analysis and interpretation at different scales of resolution and complexity.

Electron tomography (ET), which combines electron microscopy with the power of three-dimensional (3D) imaging, is the leading technique to elucidate the molecular architecture of biological specimens in a close-to-native state [1-3]. ET produces extremely noisy and low contrast 3D density maps (known as "tomograms" in the field). The poor signal-to-noise ratio (SNR) severely hinders visualization and interpretation. Sophisticated filtering techniques are thus indispensable [4]. Similar filtering needs arise in other bioimaging modalities (e.g. [5-8]).

Noise reduction is paramount for proper interpretation or post-processing of multidimensional images in bioimaging in general, and electron tomography in particular. Standard linear filtering techniques based on local averages or Gaussian kernels succeed in reducing the noise, but at the expense of blurring edges and features [4,9]. Anisotropic nonlinear diffusion (AND) is currently one of the most powerful noise reduction techniques [10]. It achieves feature preservation and enhancement as the strength and direction of the smoothing are adaptively tuned to the local structures [11,12]. However, AND may be intensive in terms of computation time and memory consumption [13] and, moreover, there is need for tuning their parameters, which may be certainly far from trivial. These drawbacks have led to the proposal of other simpler, more practical, but less powerful, methods like iterative median filtering [14], or attempts for automated parameter tuning [15].

TOMOBFLOW is a program for noise reduction with feature-preserving capabilities based upon geometric flow, particularly the so-called Beltrami flow. The fact that this approach is parameter-free is one of its main advantages and makes it user-friendly. Therefore, TOMOBFLOW combines the power of diffusion-based noise filtering approaches with the easiness from the user's point of view. Furthermore, the program has been implemented efficiently in order to minimize the memory requirements and reduce the computation time.

## Implementation

**Figure 1:**
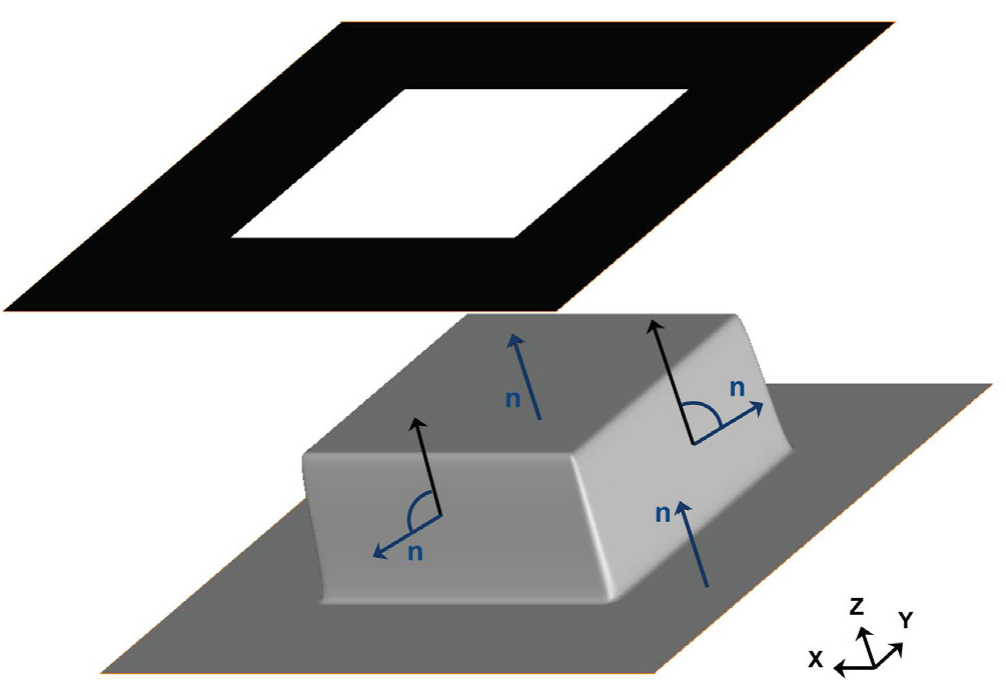
**Principles of Beltrami flow**. An image *I*(*x, y*) made up of a white square over black background (top) is viewed as a surface *S *= (*x, y, I*(*x, y*)) in a 3D space (bottom). The edges are seen as cliffs in the **Z **direction. At each point of the surface, the projection of the normal **n **(arrows in blue) to the **Z **direction (arrows in black) is the edge indicator  in Equation (1), yielding little value at sharp edges. In uniform areas, however, the normal to the surface **n **runs parallel to **Z **and the projection thus yields maximum value.

Several approaches for noise reduction in multidimensional image processing are based on considering images as maps that are embedded into a higher dimension and that flow towards minimal surfaces [16]. In these approaches, a 2D image is considered as a 2-manifold embedded in 3D, i.e. the image *I*(*x, y*) is regarded as a surface *S *= (*x, y, I*(*x, y*)) in a 3D space (see Figure 1). Similarly, a 3D volume *I*(*x, y, z*) is considered as a 3-manifold embedded in a 4D space *S *= (*x, y, z, I*(*x, y, z*)). Embedding the multidimensional image into a higher dimension allows the use of powerful differential geometry operators [16].

The Beltrami flow is an efficient geometric diffusion flow approach that aims to minimize the area of the image manifold, driving the flow towards a minimal surface solution while preserving edges. The Beltrami flow is formulated as follows [17]:

(1)

where *I*_*t *_= ∂*I*/∂*t *denotes the derivative of the image density *I *with respect to the time *t*; ▽**I **is the gradient vector, that is ▽**I **≡ (*I*_*x*_, *I*_*y*_) for 2D images whereas ▽**I **= (*I*_*x*_, *I*_*y*_, *I*_*z*_) for 3D volumes, being *I*_*x *_= ∂*I/∂x *the derivative of *I *with respect to *x *(similar applies for *y *and *z*); div is the divergence operator, defined for a vector function **f **= (*f*_*x*_, *f*_*y*_, *f*_*z*_) as div(**f**) = ∂*f*_*x*_/∂*x *+ ∂*f*_*y*_/∂*y *+ ∂*f*_*z*_/∂*z*. Finally, *g *denotes the determinant of the first fundamental form of the surface, which is *g *= 1 + |▽**I**|^2^.

The term *g *comes from an induced metric for the Euclidean (*n *+ 1)-D space where the density of a *n*-D image is embedded in the (*n *+ 1)-th dimension [16] (with *n *= 2 for 2D images and *n *= 3 for 3D volumes). This *g *provides the measure of the area expansion between the image domain *I *and the surface domain *S*, and thus plays an important role to drive the flow towards a surface with the least area.

Moreover, the term  in Equation (1) acts as an edge indicator since it is proven to be the projection of the normal-to-the-surface to the vector representing the (*n *+ 1)-th dimension [16] (see Figure 1). Therefore, the Beltrami flow is a selective noise filtering method that preserves edges as minimizes diffusion at and across edges whereas it applies extensive diffusion elsewhere [17].

In TOMOBFLOW, the implementation of the partial differential equation derived from Equation (1) is based on finite differences, using an Euler forward difference approximation for *I*_*t *_and central differences to approximate the spatial derivatives (for brevity, only the numerical approximation for the 2D case is shown):

(2)

where *I*^*k *^is the image in the *k*-th iteration, *h*_*t *_is the time step (for stability, the maximum value is the inverse of the squared number of dimensions, i.e. 0.25 for 2D images), *I*_*x *_is the derivative with respect to *x *(similar applies for *y*), *I*_*xx *_is the second order derivative with respect to *x *(similar applies for *y*) and *I*_*xy *_is the mixed second order partial derivative with respect to *x *and *y*. The derivatives are computed from the image in the previous iteration *I*^*k*-1^, and are numerically approximated by central differences, as shown for *x *(similar applies for *y*):

(3)

(4)

(5)

where *i, j *are the indices of the pixel. TOMOBFLOW has the option of applying a slight Gaussian filtering (standard deviation typically in [0.5,1.0]) to the input dataset. This initial Gaussian filtering is employed for regularization purposes to yield better estimates of the derivatives, as commonly used in other diffusion approaches [10].

**Figure 2:**
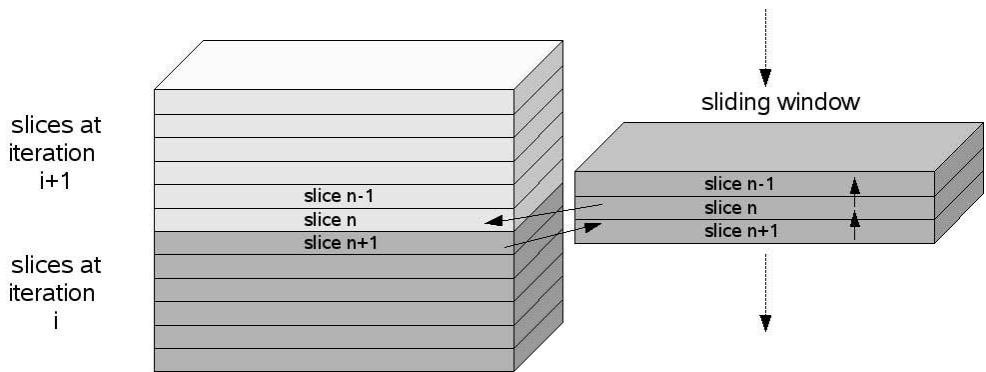
**Sliding window for processing of 3D volumes**. The sliding window keeps the data needed for the processing of the current slice. This allows TOMOBFLOW to allocate memory only for one copy of the dataset, which is progressively updated during the processing. The solid lines show the information transfer during the processing of the slice n: (1) the slice n+1 from the current volume is got from the volume, (2) the processing of the slice n is carried out using only the data in the sliding window, (3) the processed version of the slice n is updated and stored in the volume and (4) the slices in the sliding window are pushed backward to make space for a new slice coming from the volume. The dotted lines show how the sliding window is pushed forward for the processing of a new slice. The working principle of the sliding window for processing 2D images is similar.

An efficient implementation has been carried out using single processor optimization [18] to reduce memory and time consumption. Only one copy of the input dataset, which is progressively updated during the processing, is held in memory. A sliding window (3 slices for 3D volumes, 3 rows for 2D images) is used to keep the data needed for the current slice/row in order to avoid overwriting. Figure 2 illustrates how the sliding window is used during the processing of 3D volumes. This optimization allows processing of huge datasets, as commonly found in ET, on computers with modest amounts of memory.

To make it suitable for bioimaging in general, TOMOBFLOW is capable of dealing with most image formats in electron microscopy (e.g. EM, MRC, Spider), in other microscopies (e.g. Biorad) and general formats (e.g. TIF, JPG, PNG) by using the Bsoft library [19]. Furthermore, TOMOBFLOW is also able to filter 3D volumes, individual 2D images, or stacks of 2D images. Finally, it is available for most Unix platforms, including OS X and Windows (under cygwin). The command line user interface follows the Unix-style and the options follow the conventions of Bsoft [19]. A comprehensive documentation is provided at the website .

## Results

The performance of TOMOBFLOW is illustrated with its application to a number of experimental datasets obtained from electron tomography. Tomograms (3D volumes) of (a) spiny dendrite, (b) algae chloroplast, (c) mitochondrion, (d) small unilamellar liposomes with integrin, (e) vaccinia virion and (f) human immunodeficiency virions (strain HIV-1) were tested. Different contrast and signal-to-noise ratio were present in those datasets as they were obtained by using different preparation techniques. The specimens in (a-c) were stained before imaged, hence their much better contrast in the original dataset compared to the other specimens in (d-f), which were imaged while frozen in close-to-native conditions without stain. The datasets in (a, b) were taken from the Cell Centered Database [20,21] (accession codes 13 and 3408, respectively). The datasets in (d, f) were taken from the Electron Microscopy Data Bank (EMDB) at the European Bioinformatics Institute [22,23] (accession codes 1487 and 1155, respectively). The Vaccinia virus dataset was obtained from a previous work [24]. The mitochondrion dataset was kindly provided by Dr. G. Perkins. In order to compare TOMOBFLOW with other comparable standard (isotropic) nonlinear noise reduction technique, the datasets were also subjected to iterative median filtering [14] as implemented in Bsoft [19,25]. This method is getting increasing interest in the electron microscopy field [8,25-27]. The standard number of three iterations was used for all the experiments carried out in this work where the iterative median filtering was involved. For TOMOBFLOW, a number of iterations between 50 and 150 were used, which yielded a satisfactory level of smoothness for the background of the datasets. A Gaussian filtering with standard deviation of 1 was used to regularize the derivative estimation in TOMOBFLOW. For the results obtained with TOMOBFLOW, the median filtering was not used prior to the iterations of the Beltrami flow.

**Figure 3:**
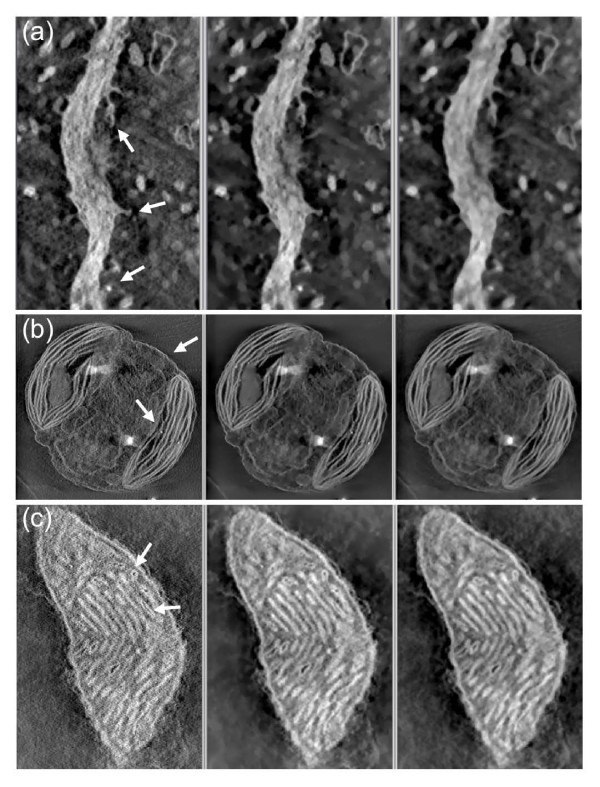
**Filtering results on the tomograms of stained specimens**. (a) spiny dendrite; (b) algae chloroplast; (c) mitochondrion. The original tomograms (left), the results with TOMOBFLOW (centre), and the results with three iterations of the median filtering (right) are shown. Only a representative slice of the tomograms is presented. The number of iterations of TOMOBFLOW were (a) 150, (b) 150 and (c) 70. The datasets (a) and (b) were taken from the Cell Centered Database (accession codes 13 and 3408, respectively). The dataset (c) was kindly provided by Dr. G. Perkins (National Center for Microscopy and Imaging Research-NCMIR, UCSD, USA). In the three datasets, the results with TOMOBFLOW have the background particularly flat with respect to the original tomogram, and also with respect to the results with median filtering. Moreover, TOMOBFLOW outperforms the median filtering in the preservation of the structural features. The arrows point to areas where the sharpness of the features is especially apparent after the processing with TOMOBFLOW.

Figure 3 shows the effects of noise reduction with TOMOBFLOW and the iterative median filtering on the tomograms obtained from the stained specimens: spiny dendrite, algae chloroplast, mitochondrion, respectively. TOMOBFLOW and the median filtering succeed in reducing the noise and preserving details. However, the results obtained with TOMOBFLOW clearly show a background substantially smoothened while showing more sharpness in the structural features of interest. The results provided by the median filtering are not that sharp and the background still contains some noisy texture.

**Figure 4:**
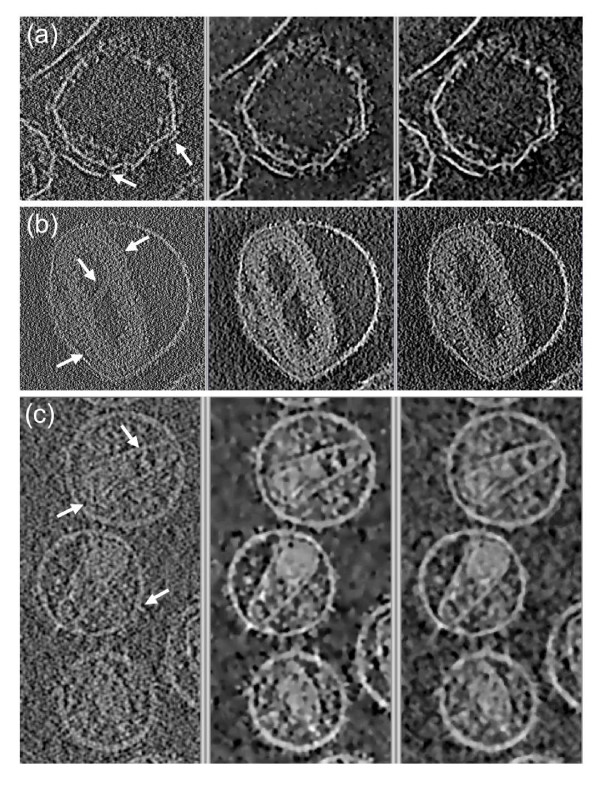
**Filtering results on the tomograms of unstained specimens**. (a) small unilamellar liposomes with integrin; (b) Vaccinia virion, (c) HIV-1 virions. The original tomograms (left), the results with TOMOBFLOW (centre), and the results with three iterations of the median filtering (right) are shown. Only a representative slice of the tomograms is presented. The number of iterations of TOMOBFLOW were (a) 100, (b) 50 and (c) 70. The datasets (a) and (c) were taken from the Electron Microscopy Data Bank (EMDB) at the European Bioinformatics Institute (accession codes 1487 and 1155, respectively). The dataset (b) comes from a previous work [24]. The behaviour of the methods are similar to that shown for unstained specimens. In general, TOMOBFLOW smoothens the background better than the median filtering and allows better identification of fine structural details (see for instance the areas pointed by the arrows, e.g. the spikes of the core of the Vaccinia virion or the membranes of the HIV-1 virions).

**Figure 5:**
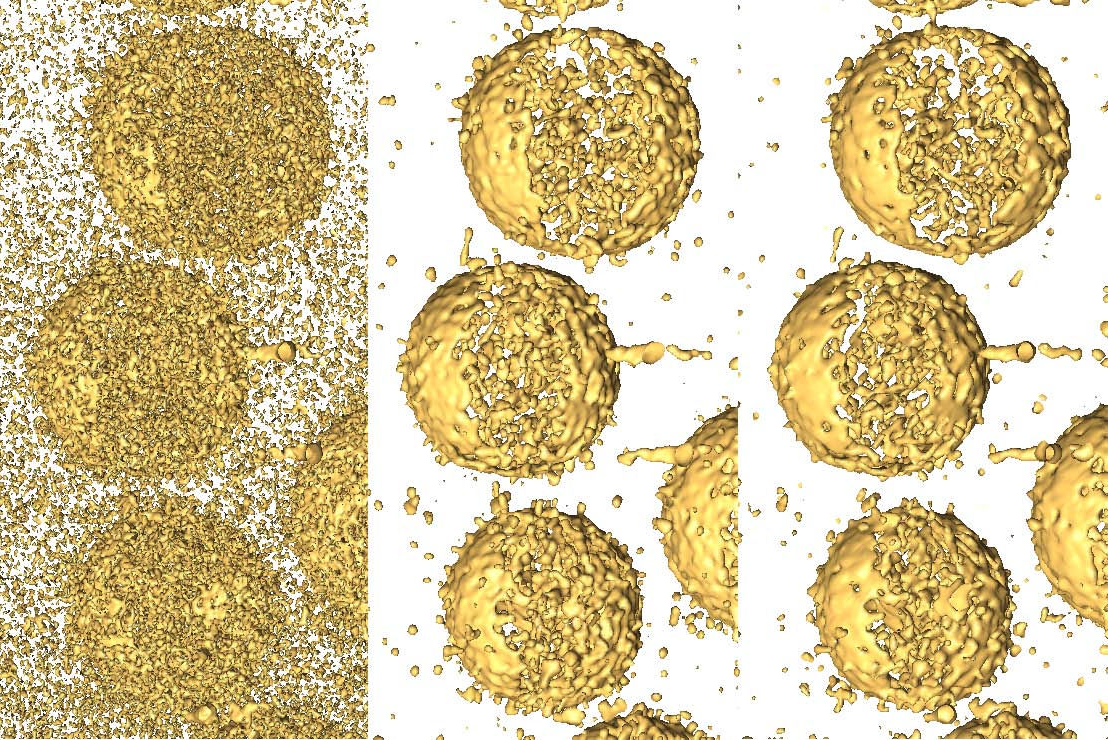
**Isosurface of the tomograms of unstained HIV-1 virions**. From left to right, the 3D visualization of the original tomogram of unstained HIV-1 virions and the denoised versions with TOMOBFLOW (70 iterations) and the iterative median filtering (3 iterations) are shown. A slice of each tomogram was previously shown in Figure 4c. Both filtering methods allow the 3D inspection of the dataset, though TOMOBFLOW preserves more details at the membranes of the virions.

Figure 4 shows the results with the datasets of the unstained specimens: small unilamellar liposomes with integrin, vaccinia virion and HIV-1 virions, respectively. The behaviour of the methods is similar to that shown with stained specimens. Both methods yield datasets that overcome the extremely low contrast and signal-to-noise ratio, thereby facilitating the interpretation. However, the results provided by TOMOBFLOW have their background further smoothened and the structural features are better preserved. For a more exhaustive analysis of the performance of TOMOBFLOW, a representative electron cryotomography dataset was selected, HIV-1 [28] (emd-1155), which was also used elsewhere to illustrate the performance of the iterative median filtering [14]. Figure 5 shows the appearance of the tomograms (original and denoised with TOMOBFLOW and the median filtering) when isosurfaced. Both methods allow a clear visualization of the virions, with similar levels of residual noise. However, a noticeable difference is seen at the membranes of the virions. They appear quite flat with the median filtering whereas more corrugations and details are seen with TOMOBFLOW, which is indicative of better structural preservation of the latter method. This effect can also be seen on the slices shown in Figure 4c.

**Figure 6:**
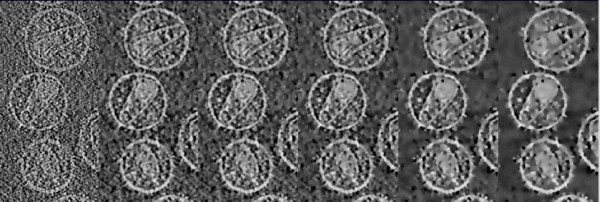
**Evolution as a function of the iterations**. From left to right, the original HIV-1 tomogram and the results with TOMOBFLOW at 10, 25, 50, 100 and 150 iterations are shown. The background is progressively flattened with the iterations whereas the structural features remain sharp. At high number of iterations, some edges begin to look blurred.

The evolution of the denoising with the iterations was then studied on the HIV-1 dataset. Figure 6 shows the result of TOMOBFLOW after 10, 25, 50, 100 and 150 iterations. It is clearly observed that the background is progressively flattened whereas the structural features are well preserved in general. However, the decrease in resolution at high number of iterations is expected to blur some details of interest. For instance, at 150 iterations some edges begin to look blurred. A more objective assessment of these results was carried out based on SNR (signal-to-noise ratio) measures, as defined in [29]: SNR = (*I*_*s *_- *I*_*b*_)/*σ*_*b*_, where *I*_*s *_and *I*_*b *_denote the average intensity in the structure of interest and in the background, respectively, and *σ*_*b *_is the standard deviation of the background. This SNR metric is similar to the contrast-to-noise ratio (CNR) used in other disciplines [30]. The background in this tomogram was determined based on the isosurfacing thresholds (calculated according to the optimal thresholding algorithm in [24]) used in Figure 5, and refined afterwards by means of some morphological operators applied in this sequence: flooding, dilation (2 cycles) and erosion (2 cycles). This background was used for computing all the SNR measures presented in this work. Table 1 summarizes the SNR results of the tomograms shown in Figure 6. The SNR measures quantitatively reflect the effects of the denoising seen in the visual results in Figure 6. The SNR for the original tomogram was 1.23. TOMOBFLOW outperforms the median filtering (SNR 2.63) at 50 or more iterations (SNR ≥ 2.79). The SNR was also computed for 200, 250 and 300 iterations of TOMOBFLOW (visual results not shown in Figure 6). As reflected by the SNR metric, the results begin to degrade at a number of TOMOBFLOW iterations between 150 and 200. Therefore, the SNR metric acts as an indicator of the degradation with high number of iterations.

**Table 1:** Effect of the denoising on the SNR of the HIV-1 dataset

**Tomogram**	**SNR**
Original	1.23
Median filtering	2.63
TOMOBFLOW 10 it.	2.31
TOMOBFLOW 25 it.	2.48
TOMOBFLOW 50 it.	2.79
TOMOBFLOW 100 it.	3.35
TOMOBFLOW 150 it.	3.55
TOMOBFLOW 200 it.	3.38
TOMOBFLOW 250 it.	3.04
TOMOBFLOW 300 it.	2.67

The SNR was computed for the original dataset and the denoised versions. For the median filtering, the standard of three iterations were used. For TOMOBFLOW, several tests with different iterations (from 10 to 300) were performed in order to study the evolution with time and to identify the decrease in resolution at high number of cycles.

The SNR metric was also used to assess the results shown in Figure 4c. The result from 70 iterations of TOMOBFLOW yielded SNR 3.03, higher than the result from the median filtering (SNR 2.63). These measures complement and confirm the visual results shown in Figure 4c. For comparison, the SNR was computed for a denoised version of the tomogram with anisotropic nonlinear diffusion, which is the leading denoising method in the field. The package TOMOAND  was used [4,11] with the automated parameter tuning activated [15]. The number of iterations (70) and the initial Gaussian filtering (std.dev.1) was set up as with TOMOBFLOW. The SNR of the TOMOAND-denoised tomogram turned out to be 4.11. Therefore, AND is superior to TOMOBFLOW, though at the expense of higher computation time and memory consumption. This behaviour was expected because the Beltrami flow is an isotropic nonlinear method and thus it is not equipped with the enhancement capabilities of anisotropic nonlinear methods, hence these two methods are not directly comparable. 

TOMOBFLOW and the iterative median filtering were also compared in terms of computation time. The average time per iteration was computed in both methods (in a standard computer based on Intel Core 2 processor 2.4 GHz running under linux) and the ratio between both was then calculated. For the six datasets, which had very different sizes (from 14 MB to 390 MB), it turned out that a single iteration in the median filtering took around 20 times more than a single iteration of TOMOBFLOW, regardless of the data size. As the number of iterations of TOMOBFLOW is usually between 50 and 150, this involves that the computation times for both methods are of the same order of magnitude (1–3 minutes for the datasets and the computer tested here). As far as memory consumption is concerned, TOMOBFLOW only used space for one copy of the dataset, as described above. It thus required half the amount of memory allocated by the median filtering (two copies of the volume) as implemented in Bsoft.

## Discussion

TOMOBFLOW allows efficient noise reduction with levels of background smoothing and feature preservation better than other comparable standard nonlinear filtering methods. TOMOBFLOW applies an isotropic nonlinear filtering method based on the Beltrami flow, which tunes the strength of the smoothing according to a local edge indicator. In contrast to anisotropic nonlinear filtering (e.g. AND), there is no enhancement of features since the direction of the smoothing is not tuned. Therefore, it must not be expected that TOMOBFLOW will outperform AND. In this regard, the comparison with AND carried out in this work suggests that the method based on the Beltrami flow lies between the median filtering and the AND methods.

The main advantage of the method implemented in TOMOBFLOW stems from the fact that there is no need for complicated parameter tuning. Nevertheless, it is indeed an iterative method and one thus needs to specify a number of iterations. But this does not pose a serious inconvenience as the program easily allows an experiment to be continued with further iterations, if necessary. On the other hand, there has been intense investigation on objective stopping criteria for iterative noise reduction methods (e.g. [4,11]). However, none of the proposed criteria have turned out to be generally applicable and the number of iterations still remains to be fixed subjectively by visual inspection of the results (e.g. [24,31]).

On the other hand, the computational burden involved by sophisticated diffusion-based filtering methods precludes their integration on interactive environments [32]. The fact that the method implemented in TOMOBFLOW is not computationally expensive along with the optimized implementation in terms of memory consumption makes this filtering method very appropriate to be embedded into interactive packages [32,33].

## Conclusion

TOMOBFLOW allows efficient noise filtering of datasets with preservation of the features of interest, thereby yielding data better suited for post-processing, visualization and interpretation. The program is versatile to deal with different types and formats of multidimensional images produced by bioimaging techniques.

## Availability and requirements

**Project name**: TOMOBFLOW

**Project home page**: 

**Operating system(s)**: Unix-based (linux, OS X, cygwin under Windows).

**Programming language**: C.

**Other requirements**: none.

**License**: public domain binaries.

**Any restrictions to use by non-academics**: none.

## Authors' contributions

JJF conceived and designed the work, developed the program, carried out the experiments, interpreted the resulting data and wrote the manuscript.
